# Efficacy of continuous positive airway pressure on TNF-α in obstructive sleep apnea patients: A meta-analysis

**DOI:** 10.1371/journal.pone.0282172

**Published:** 2023-03-23

**Authors:** Yong Luo, Fa-Rong Zhang, Jun-Lin Wu, Xi-Jiao Jiang

**Affiliations:** 1 Department of Otorhinolaryngology, The First People’s Hospital of Jiangxia District, Union Jiangnan Hospital Huazhong University of Science and Technology, Wuhan, Hubei, China; 2 Department of Endocrinology, Fifth Hospital in Wuhan, Wuhan, Hubei, China; University of Catania, ITALY

## Abstract

**Background:**

Tumor necrosis factor-α (TNF-α) is an important mediator of the immune response. At present, the improvement of TNF-α after continuous positive airway pressure (CPAP) treatment of obstructive sleep apnea-hypopnea syndrome (OSAHS) is still controversial.

**Methods:**

We conducted a systematic review of the present evidence based on a meta-analysis to elucidate the effects of TNF-α on OSAHS after CPAP treatment.

**Results:**

To measure TNF-α, ten studies used enzyme-linked immunosorbent assay (ELISA), and one used radioimmunoassay. The forest plot outcome indicated that CPAP therapy would lower the TNF-α levels in OSAHS patients, with a weighted mean difference (WMD) of 1.08 (95% CI: 0.62–1.55; *P* < 0.001) based on the REM since there is highly significant heterogeneity (*I*^*2*^ = 90%) among the studies. Therefore, we used the subgroup and sensitivity analyses to investigate the source of heterogeneity. The findings of the sensitivity analysis revealed that the pooled WMD ranged from 0.91 (95% CI: 0.52–1.31; *P* < 0.001) to 1.18 (95% CI: 0.74–1.63; *P* < 0.001). The findings were not influenced by any single study. Notably, there was homogeneity in the Asia subgroup and publication year: 2019, implying that these subgroups could be the source of heterogeneity.

**Conclusion:**

Our meta-analysis recommends that CPAP therapy will decrease the TNF-α level in OSAHS patients, but more related research should be conducted.

## Introduction

Obstructive sleep apnea-hypopnea syndrome (OSAHS) is characterized by repeated episodes of partial or complete upper airway obstruction during sleep, resulting in chronic intermittent hypoxemia, excessive daytime sleepiness, irregular snoring at night, and increased nocturia. It is recognized as a new controllable risk factor for cerebrovascular and cardiovascular disease [1, 2]. Numerous studies reported that 3–9% of females and 10–17% of males have an apnea–hypopnea index (AHI) ≥ 15/h, which is present in approximately one billion people aged 30 to 69 years globally [3, 4]. Repeated hypoxic events and concomitant sleep disruption cause ventilatory instability, oxidative stress, inflammation, and disruption of vascular function [5]. The gold-standard treatment for OSAHS is a device that provides continuous pressure on the upper airway, called continuous positive airway pressure (CPAP), which can correct intermittent hypoxemia, reduce vascular endothelial dysfunction, and decreases ventilatory responsiveness to hypoxia [6]. Moreover, CPAP has a consistent ancillary effect, which includes intermittent hypercapnia, sleep fragmentation, repetitive increases in negative intrathoracic pressure, sympathetic nerve activity surge, and blood pressure (BP) [7–9].

Tumor necrosis factor-α (TNF-α) is a multidirectional pro-inflammatory cytokine secreted by various cells, including adipocytes, activated monocytes, macrophages, B cells, T cells, and fibroblasts. TNF-α is an important mediator of the immune response. Chronic inflammatory injury involving numerous inflammatory factors such as TNF-α, C-reactive protein, interleukin-6 (IL-6), and interleukin-8 (IL-8) is the leading cause of cardiovascular and cerebrovascular complications [10, 11]. In OSAHS patients, an increase in serum TNF-α affects lipid metabolism and energy consumption, resulting in weight gain and metabolic disorders [12]. Concurrently, TNF-α and other pro-inflammatory factors can also regulate the hypothalamus and hippocampus during sleep, causing structural sleep disturbances [13]. Many studies currently revealed that appropriate CPAP treatment could improve arterial inflammation and metabolic status in OSAHS patients [14]. However, some studies indicated that CPAP treatment had no significant effect on the inflammatory factor TNF-α [15]. The improvement of TNF-α after CPAP treatment of OSAHS is still controversial. Borges *et al*. [15] revealed no significant differences in the levels of oxidative stress and inflammation markers in OSAHS patients after eight weeks of CPAP. While Wang *et al*. [16] demonstrated that the CPAP therapy significantly reduced the incidence of all arrhythmia in OSAHS patients, TNF-α was significantly lower in the CPAP group than in the sham-CPAP group. Despite numerous original studies on CPAP and TNF-α in OSAHS patients, there is a lack of comprehensive scientific evidence. The question in the present analysis is: "Does CPAP treatment reduce TNF-α levels in OSAHS patients?" Therefore, we conducted a meta-analysis-based systematic review of the current evidence to elucidate the effects of TNF-α on OSAHS after CPAP treatment.

## Methods

### Search strategy

To determine potential applicable original articles, a thorough search in the PubMed, EMBASE, and Web of Science databases up to January 2, 2022, using the following words: "CPAP", "continuous positive airway pressure", "tumor necrosis factor-α", "TNF-α", "OSA", "obstructive sleep apnea-hypopnea syndrome", "obstructive sleep apnea" and "OSAHS". We first screened the article’s title and abstract and reviewed the included research reference lists to find additional relevant articles.

### Inclusion and exclusion standard

The present study had no national limitations. All OSAHS patients received CPAP treatment; plasma TNF-α was measured before and after the CPAP; studies were limited to humans, published in English, and included detailed raw data. Cancers and other illnesses that could be associated with TNF-α were not included. The present study excluded repeated studies, letters, case reports, abstracts, and comments. We extracted the first author’s name and publication year, sample size, country, CPAP time, AHI, BMI, and age from the final included studies. After removing duplicates, two independent reviewers (ZFR and LY) assessed the studies against the inclusion and exclusion criteria. The third author (JJX) resolves conflicts.

### Statistical analysis

The value of the *I*^*2*^ index determines heterogeneity. *I*^*2*^ values of 75–100%, 50–75%, 25–50%, and < 25% indicated high, medium, and low heterogeneity and homogeneity. If the *I*^*2*^ value is > 50%, the random effect model (REM) is used; otherwise, the fixed effect model (FEM) is used. The weighted mean difference (WMD) of TNF-α levels was calculated for each study. The sensitivity analysis was repeated several times to evaluate the impact of each study in the analysis, each time excluding a different individual study. By excluding different individual studies each time, the sensitivity analysis was repeated to evaluate the impact of each study in the analysis.

## Results

### Article features

**Fig 1:**
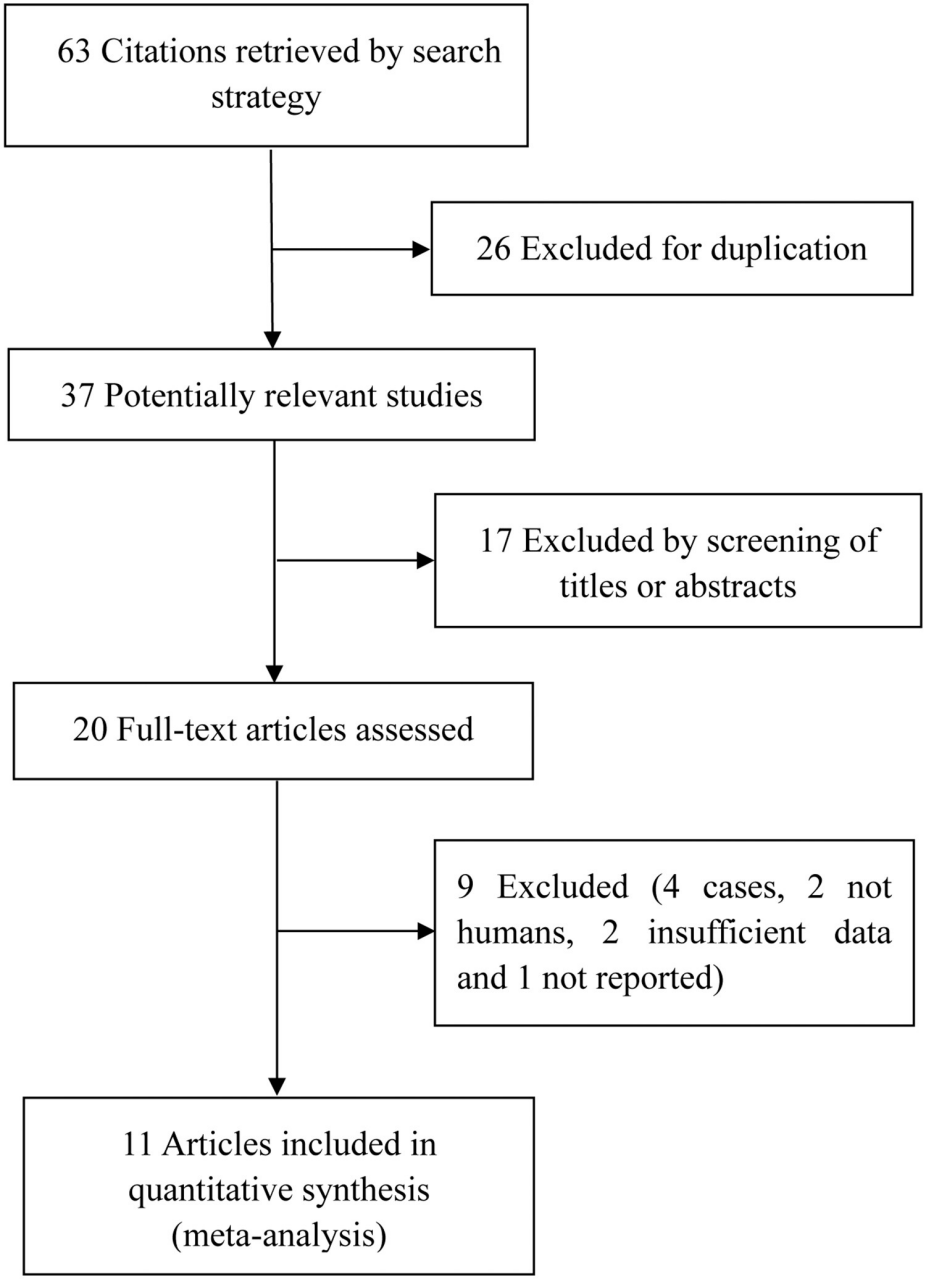
Selection process for studies included in the meta-analysis.

Sixty-three articles were identified as relevant. The removal of repeated studies reduced the number of studies to thirty-seven. The remaining thirty-seven studies were screened, resulting in the exclusion of seventeen studies. After reviewing the full text of the twelve studies, nine articles were excluded. Finally, eleven articles were included in the meta-analysis [15, 17–26]. Fig 1 depicts the literature retrieval procedure. Meanwhile, Table 1 presents the study data. Three studies came from Asia, five from Europe, and three from America. All the articles had NOS scores of five or above, indicating their high quality. Moreover, ten articles were observational studies, while one was a randomized controlled trial [18]. To measure TNF-α, ten studies used enzyme-linked immunosorbent assay (ELISA), and one used radioimmunoassay.

**Table 1:** Description of included studies.

Study	Country	N	Assay	Age	CPAP time	NOS	AHI (Pre/Post CPAP)	BMI (Pre/Post CPAP)
Borges et al [15], 2019	Brazil	15	ELISA	53 ± 10	12-month	5	NA	31 ± 6/NA
Vicente et al [17], 2016	Spain	89	ELISA	44 (36–56)	12-month	6	28 (12–44)/NA	29.1 (27.1–33.9)/NA
Carneiro et al [18], 2009	Brazil	7	ELISA	40.1±2.8	6-month	5	91.0 ± 9.7/15.3 ± 11.1	46.1 ± 2.8/46.8 ± 2.6
Karamanl et al [19], 2012	Turkey	35	ELISA	NA	3-month	5	45.6±22.1/3.8±2.9	NA
Minoguchi et al [20], 2004	Japan	12	ELISA	NA	1-month	7	NA	NA
Morohunfolu et al [21], 2013	USA	29	Radioimmunoassay	54.5±8.9	2-month	6	32.2±13.1/NA	31.1±5.7/NA
Rodriguez et al [22], 2019	Spain	120	ELISA	60.0 (53.0–67.0)	12 weeks	7	35.9 (24.2–50.1)/NA	33.5 (28.8–37.1)/NA
Nural et al [23], 2013	Turkey	25	ELISA	50.88±7.87	1-month	5	44.98±24.48/7.06±6.09	NA
Tamaki et al [24], 2009	Japan	33	ELISA	NA	3-month	7	NA	NA
Ryan et al [25], 2006	Ireland	49	ELISA	NA	6 weeks	7	NA	NA
Jiang et al [26], 2017	China	92	ELISA	NA	6-month	7	NA	NA

NA: not given, CPAP: continuous positive airway pressure, AHI: apnea hyponea index, BMI: body mass index, NOS: Newcastle-Ottawa scale.

### Pooled analysis

**Fig 2:**
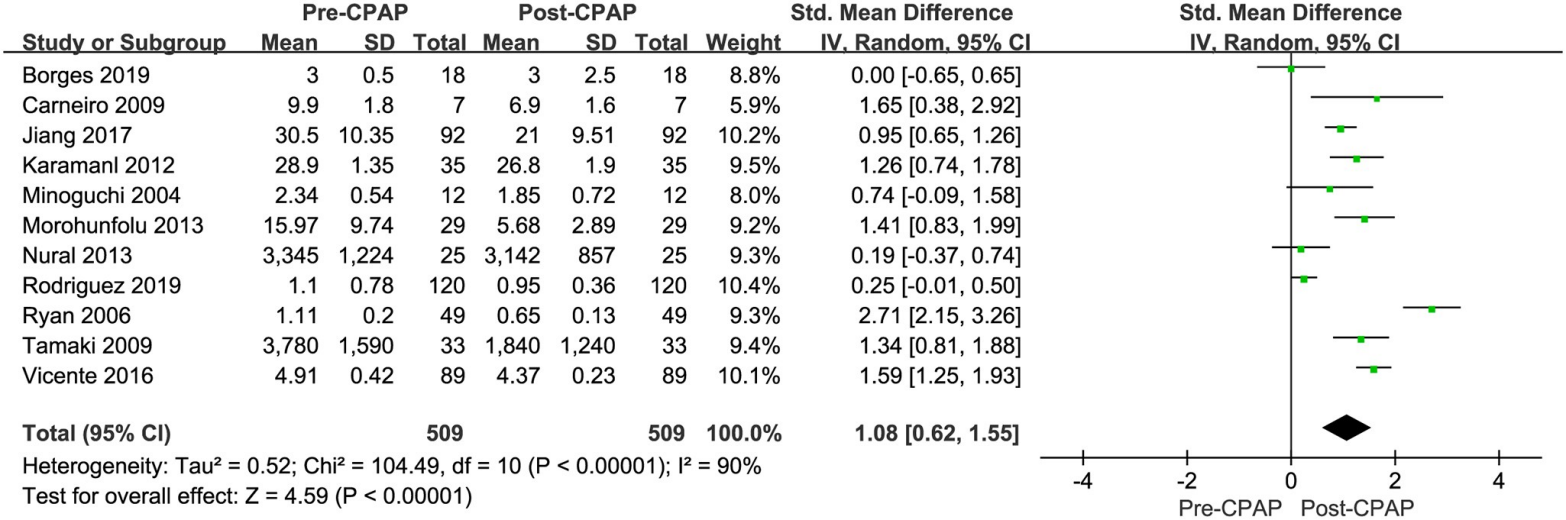
The forest plot outcome indicated that the CPAP therapy will not change the TNF-α level in OSAHS patients.

The forest plot outcome indicated that CPAP therapy would lower the TNF-α levels in OSAHS patients, with a WMD of 1.08 (95% CI: 0.62–1.55; *P* < 0.001) based on the REM since there is highly significant heterogeneity (*I*^*2*^ = 90%) among the studies Fig 2. Therefore, we used the subgroup and sensitivity analyses to investigate the source of heterogeneity. The findings of the sensitivity analysis revealed that the pooled WMD ranged from 0.91 (95% CI: 0.52–1.31; *P* < 0.001) to 1.18 (95% CI: 0.74–1.63; *P* < 0.001). The findings were not influenced by any single study.

### Subgroup analysis

Change in TNF-α levels in OSAHS patients was also investigated using subgroup analysis. Patient number and CPAP duration time were also significant in the region subgroup, and the complete information is presented in Table 2. Notably, there was homogeneity in the Asia subgroup and publication year: 2019, implying that these subgroups could be the source of heterogeneity. Begg’s test (*P* = 0.075) and Egger’s test (*P* = 0.154) indicated a significant negative publication bias.

**Table 2:** Results of subgroup analysis among included studies.

Subgroup	Studies included (N)	Heterogeneity		Pooled WMD	Pooled *P* value
		*I*^*2*^ (%)	*P* value		
Asia	3	1	0.36	1.02 (0.76–1.28)	<0.001
Europe	5	96	<0.001	1.19 (0.32–2.06)	0.006
America	3	83	0.003	0.92 (-0.12–2.04)	0.08
CPAP>3 months	6	76	0.0007	1.11 (0.69–1.53)	<0.001
CPAP<3 months	5	94	<0.001	1.05 (0.10–2.01)	0.03
Number<15	2	27	0.24	1.06 (0.21–1.91)	0.01
Publication year: 2019	2	0	0.49	0.21 (-0.02–0.45)	0.08

## Discussion

Our meta-analysis suggests that CPAP therapy reduces TNF-α levels in OSAHS patients. Simultaneously, the present study illustrates high heterogeneity. In addition, subgroup analysis was performed to investigate the differences between TNF-α level and OSAHS. Furthermore, sensitivity analysis revealed that the overall results remained unchanged when any single study was excluded or REM was converted to FEM. Therefore, we believe the data obtained from the present study are reliable.

Long-term CPAP therapy decreases circulating levels of TNF-αin OSA patients [23], which is consistent with our findings, as CPAP therapy lowers TNF-α levels in OSAHS patients. Neutrophils and monocytes secreted cytokines and proteins stimulated by TNF-α during nocturnal hypoxia. Various studies in animal models of OSAHS have indicated that TNF-α can stimulate cells to produce IL-1 and IL-17, which ultimately leads to the recruitment of neutrophils and plays an important role in the deterioration of OSA at night [27]. Similarly, Minoguchi *et al*. [20] described that deterioration of sleep quality due to repeated apnea-associated hypoxia is associated with increased TNF-α production in OSA patients. In addition, NF-κB signaling is important in stimulate TNF-α in OSA [20]. Studies indicated that TLR2/TLR4 activation had been associated with the release of TNF-αfrom monocytes in OSA patients through NF-κB signaling [21]. Moreover, body mass index (BMI) and AHI have been independently associated with systemic TNF-α production [12]. BMI was the strongest predictor of TNF-α production by monocytes, indicating that adipocytes and monocytes primarily produce TNF-α in response to hypoxia [20]. Due to insufficient data, we were unable to investigate the role of BMI in TNF-α levels in OSA patients in the present study. Because sympathetic nervous activation causes 2-adrenergic receptor-mediated leukocytosis and CPAP reduces catecholamine levels and sympathetic nerve activation, the monocytes gained before and after CPAP may represent distinct populations, illustrating the decrease in TNF-α production. Meanwhile, AHI is a predictor of inflammation in OSA patients. A significant correlation was found between AHI changes and spontaneous TNF-α production by monocytes [20]. However, due to the lack of data in the present study, we cannot investigate the role of AHI in TNF-α levels in OSA patients. So far, no meta-analysis has explored the change of TNF-α in OSA patients to strengthen the evidence. Therefore, our meta-analysis indicated that CPAP therapy reduces the TNF-α levels in OSAHS patients, which may contribute to better clinical management of OSA patients.

OSA is a risk factor for obesity and cardiovascular disease. The cytokine TNF-α gene is also linked to OSAHS susceptibility; the frequency of the ’-308A’ allele in the TNF-α gene was significantly higher in obese patients with OSA compared to obese subjects without OSA [28]. In addition, the present study found that inhibiting TNF-α activity was associated with a significant reduction in objective sleepiness in obese OSA patients. This effect is approximately three times greater than the effect of CPAP ventilation on objective sleepiness in OSA patients, indicating that pro-inflammatory cytokines promote OSA pathogenesis [29]. Therefore, early use of CPAP can reduce the level of inflammation in the OSA patient, alleviating drowsiness and lowering the morbidity and mortality from cardiovascular diseases [30]. In the present study, we also found a significant result in the subgroup analysis, implying that changes in clinical characteristics will affect changes in TNF-α levels in OSAHS patients, but further investigation is required. While we could not investigate the role of BMI and AHI in the impact of TNF-α in OSAHS patients due to a lack of data, additional correlated research on BMI and AHI stratification should be conducted.

So far, this is the first meta-analysis to determine whether OSAHS patients are related to TNF-α and CPAP treatment. Simultaneously, our study also has some limitations. First, we lack information on BMI, AHI, age, gender, and CPAP time due to a lack of data. Second, the results may be biased due to the differences in TNF-α susceptibility and measurement methods. Finally, primary prevention necessitates a large number of subjects and a more extended follow-up period, which can cause information deviation and affect the accuracy of the results.

## Conclusion

Our meta-analysis recommends that CPAP therapy decrease the TNF-α level in OSAHS patients, but more related research should be conducted.

## Supporting information

S1 Checklist PRISMA 2020 checklist. (DOCX)
