# Supplementary material for: Efficacy of continuous positive airway pressure on TNF-α in obstructive sleep apnea patients: A meta-analysis
Source: PLoS One. 2023 Mar 23;18(3):e0282172. doi: 10.1371/journal.pone.0282172 (PMC10035913; doi:10.1371/journal.pone.0282172)
Supplement: S1 Checklist — (DOCX) [file pone.0282172.s001.docx]

| **Section and Topic** | **Item #** | **Checklist item** | **Location where item is reported** |
| --- | --- | --- | --- |
| **TITLE** | | |  |
| Title | 1 | Efficacy of Continuous Positive Airway Pressure on TNF-α in Obstructive Sleep Apnea Patients: A Meta-Analysis | 1 |
| **ABSTRACT** | | |  |
| Abstract | 2 | Background: TNF-α is an important factor that mediates the immune response. At present, the improvement of TNF-α after CPAP treatment of OSAHS is still controversial.  Methods: We conducted a systematic review of the present evidence based on a meta-analysis to elucidate the effects of TNF-α on OSAHS after CPAP treatment.  Results: The forest plot outcome indicated that the CPAP therapy will decrease the TNF-α level in OSAHS patients, with a WMD of 1.08 (95% CI: 0.62 to 1.55; P<0.001) based on the REM since as a high significant heterogeneity (I2=90%) among the studies. The results of sensitivity analysis indicated that the pooled WMD ranged from 0.91 (95% CI: 0.52–1.31; P<0.001) to 1.18 (95% CI: 0.74–1.63; P<0.001). No single study influenced the results.  Conclusion: Our meta-analysis recommend that CPAP therapy will decrease the TNF-α level in OSAHS patients, but more related researches should be conducted. | 1 |
| **INTRODUCTION** | | |  |
| Rationale | 3 | TNF-α is an important factor that mediates the immune response. At present, the improvement of TNF-α after CPAP treatment of OSAHS is still controversial. | 1 |
| Objectives | 4 | TNF-α is an important factor that mediates the immune response. At present, the improvement of TNF-α after CPAP treatment of OSAHS is still controversial. | 1 |
| **METHODS** | | |  |
| Eligibility criteria | 5 | This study had no national limitations. All OSAHS patients were given CPAP treatment; plasma TNF-α was tested before or at the end of the CPAP; studies limited to humans, published in English, detailed raw data. The possible correlative sickness that might unstable with TNF-α (such as cancers) was not included. Repeated studies, letters, case reports, abstracts, and comments were excluded from the study. Extracted the first author name and publication year, sample size, country, CPAP time, AHI, BMI and age from the final included researches. | 2 |
| Information sources | 6 | To determine potential applicable original articles, a thorough search in PubMed, EMBASE, and Web of Science databases up to January 2, 2022, applying the following words: “CPAP”, “continuous positive airway pressure”, “tumor necrosis factor-α”, “TNF-α”, “OSA”, “obstructive sleep apnea-hypopnea syndrome”, “obstructive sleep apnea” and “OSAHS”. We first screened the title and abstract of the article and also reviewed reference lists of the included researches to find additional eligible relevant articles. | 2 |
| Search strategy | 7 | To determine potential applicable original articles, a thorough search in PubMed, EMBASE, and Web of Science databases up to January 2, 2022, applying the following words: “CPAP”, “continuous positive airway pressure”, “tumor necrosis factor-α”, “TNF-α”, “OSA”, “obstructive sleep apnea-hypopnea syndrome”, “obstructive sleep apnea” and “OSAHS”. We first screened the title and abstract of the article and also reviewed reference lists of the included researches to find additional eligible relevant articles. | 2 |
| Selection process | 8 | To determine potential applicable original articles, a thorough search in PubMed, EMBASE, and Web of Science databases up to January 2, 2022, applying the following words: “CPAP”, “continuous positive airway pressure”, “tumor necrosis factor-α”, “TNF-α”, “OSA”, “obstructive sleep apnea-hypopnea syndrome”, “obstructive sleep apnea” and “OSAHS”. We first screened the title and abstract of the article and also reviewed reference lists of the included researches to find additional eligible relevant articles. | 2 |
| Data collection process | 9 | To determine potential applicable original articles, a thorough search in PubMed, EMBASE, and Web of Science databases up to January 2, 2022, applying the following words: “CPAP”, “continuous positive airway pressure”, “tumor necrosis factor-α”, “TNF-α”, “OSA”, “obstructive sleep apnea-hypopnea syndrome”, “obstructive sleep apnea” and “OSAHS”. We first screened the title and abstract of the article and also reviewed reference lists of the included researches to find additional eligible relevant articles. | 2 |
| Data items | 10a | Extracted the first author’s name and publication year, sample size, country, CPAP time, AHI, BMI and age from the final included researches. | 2 |
|  | 10b | List and define all other variables for which data were sought (e.g. participant and intervention characteristics, funding sources). Describe any assumptions made about any missing or unclear information. | 2 |
| Study risk of bias assessment | 11 | The sensitivity analysis was repeated to assess the impact of each study in the analysis by excluding different individual studies each time. | 3 |
| Effect measures | 12 | If the I2 value is greater than 50%, the random effect model (REM) is used, and if the I2 value is less than 50%, the fixed effect model (FEM) is used. For each study, the weighted mean difference (WMD) of each study of the TNF-α levels were calculated. | 3 |
| Synthesis methods | 13a | To determine potential applicable original articles, a thorough search in PubMed, EMBASE, and Web of Science databases up to January 2, 2022, applying the following words: “CPAP”, “continuous positive airway pressure”, “tumor necrosis factor-α”, “TNF-α”, “OSA”, “obstructive sleep apnea-hypopnea syndrome”, “obstructive sleep apnea” and “OSAHS”. We first screened the title and abstract of the article and also reviewed reference lists of the included researches to find additional eligible relevant articles. | 2 |
|  | 13b | NA |  |
|  | 13c | NA |  |
|  | 13d | NA |  |
|  | 13e | NA |  |
|  | 13f | NA |  |
| Reporting bias assessment | 14 | The sensitivity analysis was repeated to assess the impact of each study in the analysis by excluding different individual studies each time. | 3 |
| Certainty assessment | 15 | The sensitivity analysis was repeated to assess the impact of each study in the analysis by excluding different individual studies each time. | 3 |
| **RESULTS** | | |  |
| Study selection | 16a | Sixty-three relevant articles were determined. Removal of repeats reduced the count to thirty-seven studies. The thirty-seven studies were still screened and this resulted to ruling out of seventeen studies. Nine articles were not included afterward browsing of the full text of the twelve researches. Finally, eleven articles included the meta-analysis11, 13-22. The procedure of literature retrieval are shown in Figure 1. Meanwhile, the study information is shown in Table 1. Three studies from Asia, five studies from Europe and three study from America. The NOS scores of all articles were 5 or above which means that they are of good quality. | 3 |
|  | 16b | Sixty-three relevant articles were determined. Removal of repeats reduced the count to thirty-seven studies. The thirty-seven studies were still screened and this resulted to ruling out of seventeen studies. Nine articles were not included afterward browsing of the full text of the twelve researches. Finally, eleven articles included the meta-analysis11, 13-22. The procedure of literature retrieval are shown in Figure 1. Meanwhile, the study information is shown in Table 1. Three studies from Asia, five studies from Europe and three study from America. The NOS scores of all articles were 5 or above which means that they are of good quality. | 3 |
| Study characteristics | 17 | Sixty-three relevant articles were determined. Removal of repeats reduced the count to thirty-seven studies. The thirty-seven studies were still screened and this resulted to ruling out of seventeen studies. Nine articles were not included afterward browsing of the full text of the twelve researches. Finally, eleven articles included the meta-analysis11, 13-22. The procedure of literature retrieval are shown in Figure 1. Meanwhile, the study information is shown in Table 1. Three studies from Asia, five studies from Europe and three study from America. The NOS scores of all articles were 5 or above which means that they are of good quality. | 3 |
| Risk of bias in studies | 18 | The forest plot outcome indicated that the CPAP therapy will decrease the TNF-α level in OSAHS patients, with a WMD of 1.08 (95% CI: 0.62 to 1.55; P<0.001) based on the REM since as a high significant heterogeneity (I2=90%) among the studies. The results of sensitivity analysis indicated that the pooled WMD ranged from 0.91 (95% CI: 0.52–1.31; P<0.001) to 1.18 (95% CI: 0.74–1.63; P<0.001). No single study influenced the results. | 3 |
| Results of individual studies | 19 | The forest plot outcome indicated that the CPAP therapy will decrease the TNF-α level in OSAHS patients, with a WMD of 1.08 (95% CI: 0.62 to 1.55; P<0.001) based on the REM since as a high significant heterogeneity (I2=90%) among the studies. The results of sensitivity analysis indicated that the pooled WMD ranged from 0.91 (95% CI: 0.52–1.31; P<0.001) to 1.18 (95% CI: 0.74–1.63; P<0.001). No single study influenced the results. | 3 |
| Results of syntheses | 20a | NA |  |
|  | 20b | NA |  |
|  | 20c | NA |  |
|  | 20d | NA |  |
| Reporting biases | 21 | NA |  |
| Certainty of evidence | 22 | The forest plot outcome indicated that the CPAP therapy will decrease the TNF-α level in OSAHS patients, with a WMD of 1.08 (95% CI: 0.62 to 1.55; P<0.001) based on the REM since as a high significant heterogeneity (I2=90%) among the studies. The results of sensitivity analysis indicated that the pooled WMD ranged from 0.91 (95% CI: 0.52–1.31; P<0.001) to 1.18 (95% CI: 0.74–1.63; P<0.001). No single study influenced the results. | 3 |
| **DISCUSSION** | | |  |
| Discussion | 23a | Our meta-analysis recommend that CPAP therapy will decrease the TNF-α level in OSAHS patients, therefore, it is relative accurate to use TNF-α level as an index to evaluate the inflammation function in patients with OSAHS. Simultaneously, our study illustrates that there is high heterogeneity. In addition, subgroup analysis to investigate the differences among TNF-α level and OSAHS. Moreover, sensitivity analysis showed that the overall results remained unchanged when any single study was excluded or REM was converted to FEM. Therefore, we believe that the data obtained from our study are reliable. | 3 |
|  | 23b | So far, this is the first attempt to prove whether OSAHS patients are related to TNF-α and CPAP treatment through meta-analysis. At the same time, our research also has limitations. First, due to insufficient data, we have no information about BMI, AHI, age, gender, CPAP time, etc. Secondly, due to the different susceptibility and measurement methods of TNF-α, the results may be biased. Finally, primary prevention requires a large number of subjects and long follow-up time, which may lead to information deviation and affect the accuracy of the results. | 3 |
|  | 23c | So far, this is the first attempt to prove whether OSAHS patients are related to TNF-α and CPAP treatment through meta-analysis. At the same time, our research also has limitations. First, due to insufficient data, we have no information about BMI, AHI, age, gender, CPAP time, etc. Secondly, due to the different susceptibility and measurement methods of TNF-α, the results may be biased. Finally, primary prevention requires a large number of subjects and long follow-up time, which may lead to information deviation and affect the accuracy of the results. | 4 |
|  | 23d | Our meta-analysis recommend that CPAP therapy will decrease the TNF-α level in OSAHS patients, but more related researches should be conducted. | 4 |
| **OTHER INFORMATION** | | |  |
| Registration and protocol | 24a | NA |  |
|  | 24b | NA |  |
|  | 24c | NA |  |
| Support | 25 | NA |  |
| Competing interests | 26 | NA |  |
| Availability of data, code and other materials | 27 | NA |  |

*From:*  Page MJ, McKenzie JE, Bossuyt PM, Boutron I, Hoffmann TC, Mulrow CD, et al. The PRISMA 2020 statement: an updated guideline for reporting systematic reviews. BMJ 2021;372:n71. doi: 10.1136/bmj.n71

For more information, visit: <http://www.prisma-statement.org/>
